# Activation of the bile acid receptors TGR5 and FXR in the spinal dorsal horn alleviates neuropathic pain

**DOI:** 10.1111/cns.14154

**Published:** 2023-03-07

**Authors:** Yuning Wu, Yuxin Qiu, Minzhi Su, Linjie Wang, Qingjuan Gong, Xuhong Wei

**Affiliations:** ^1^ Department of Human Anatomy and Physiology, Zhongshan School of Medicine Sun Yat‐sen University Guangzhou China; ^2^ Department of Anesthesiology The First Affiliated Hospital, Sun Yat‐sen University Guangzhou China; ^3^ Department of Rehabilitation The Third Affiliated Hospital and Lingnan Hospital of Sun Yat‐Sen University Guangzhou China; ^4^ Department of Pain Medicine The Second Affiliated Hospital of Guangzhou Medical University Guangzhou China; ^5^ Guangdong Provincial Key Laboratory of Brain Function and Disease, Zhongshan School of Medicine Sun Yat‐sen University Guangzhou China; ^6^ Pain Research Center, Zhongshan School of Medicine Sun Yat‐sen University Guangzhou China

**Keywords:** astrocyte, bile acid, mechanical allodynia, nuclear farnesoid X receptor, spinal cord, Takeda G protein‐coupled receptor 5

## Abstract

**Aims:**

Beyond digestion, bile acids have been recognized as signaling molecules with broad paracrine and endocrine functions by activating plasma membrane receptor (Takeda G protein‐coupled receptor 5, TGR5) and the nuclear farnesoid X receptor (FXR). The present study investigated the role of bile acids in alleviating neuropathic pain by activating TGR5 and FXR.

**Method:**

Neuropathic pain was induced by spared nerve injury (SNI) of the sciatic nerve. TGR5 or FXR agonist was injected intrathecally. Pain hypersensitivity was measured with Von Frey test. The amount of bile acids was detected using a bile acid assay kit. Western blotting and immunohistochemistry were used to assess molecular changes.

**Results:**

We found that bile acids were downregulated, whereas the expression of cytochrome P450 cholesterol 7ahydroxylase (CYP7A1), a rate‐limiting enzyme for bile acid synthesis, was upregulated exclusively in microglia in the spinal dorsal horn after SNI. Furthermore, the expression of the bile acid receptors TGR5 and FXR was increased in glial cells and GABAergic neurons in the spinal dorsal horn on day 7 after SNI. Intrathecal injection of either TGR5 or FXR agonist on day 7 after SNI alleviated the established mechanical allodynia in mice, and the effects were blocked by TGR5 or FXR antagonist. Bile acid receptor agonists inhibited the activation of glial cells and ERK pathway in the spinal dorsal horn. All of the above effects of TGR5 or FXR agonists on mechanical allodynia, on the activation of glial cells, and on ERK pathway were abolished by intrathecal injection of the GABA_A_ receptor antagonist bicuculline.

**Conclusion:**

These results suggest that activation of TGR5 or FXR counteracts mechanical allodynia. The effect was mediated by potentiating function of GABA_A_ receptors, which then inhibited the activation of glial cells and neuronal sensitization in the spinal dorsal horn.

## Graphical abstract

Peripheral nerve injury increased expression of bile acid receptor TGR5 and FXR in glial cells and GABAergic neurons in the spinal dorsal horn. Activation of TGR5 or FXR alleviated the established mechanical allodynia by inhibiting the activation of glial cells and the expression of p‐ERK in the spinal dorsal horn, followed by increasing the inhibitory effect of GABAA receptor.

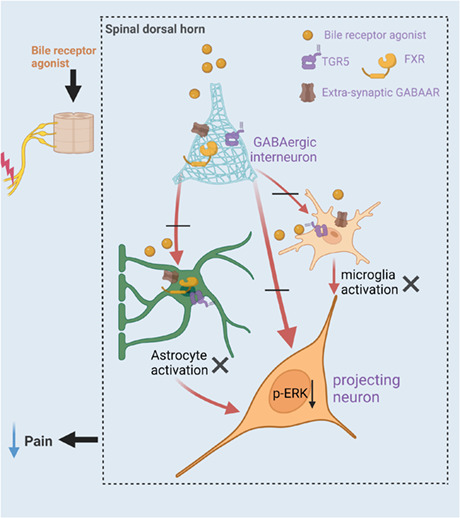

## 1 INTRODUCTION

Neuropathic pain following peripheral nerve injury is considered a disease of the nervous system.^1^ There are currently no good therapies to treat neuropathic pain, highlighting the lack of understanding of detailed mechanisms and the urgency of discovering new drug targets.

After peripheral nerve injury, spinal microglia are activated, possibly by macrophage colony‐stimulating factor‐1 (CSF‐1) and adenosine triphosphate (ATP), which are released from primary afferents, and fractalkine is released from spinal projecting neurons.^2^ Activated microglia can release inflammatory mediators, such as tumor necrosis factor α (TNF‐α), interleukin 1β (IL‐1β), and interleukin‐6 (IL‐6), to activate and alter the function of astrocytes.^3^ Activated microglia and astrocytes initiate neuroinflammation to alter the environment in which neurons live, rendering nociceptive neurons in the spinal dorsal horn more excitable and pain transmission enhanced and prolonged.^4^, ^5^ Previous studies, including ours, have shown that inhibiting the synthesis of cytokines, antagonizing their receptors, or inhibiting downstream signaling pathways, such as the ERK pathway, can alleviate the established neuropathic pain.^6^, ^7^, ^8^ Despite the above extensive studies, whether there are endogenous factors that can limit neuroinflammation during pain chronicity is still unclear

Bile acids are synthesized from cholesterol by two pathways: the classic (neutral) pathway or the alternative (acidic) pathway. The classic pathway, initiated by the rate‐limiting enzyme cytochrome P450 cholesterol 7ahydroxylase (CYP7A1), produces the majority of the bile acid pool. In the liver, primary bile acids, including chenodeoxycholic acid (CDCA) and cholic acid (CA), are synthesized from cholesterol, conjugated with glycine or taurine, secreted into the gallbladder, and transported to the intestine to be metabolized by gut bacteria. This classic pathway produces approximately 90% of bile acids synthesis. The alternative pathway, initiated by the mitochondrial cytochrome P450 sterol 27‐hydroxylase (CYP27A1), produces the remaining 10% of BA and is primarily extrahepatic, including the brain.^9^, ^10^

Bile acids can exert hormone‐like effects by integrating with their receptors, including the G‐protein‐coupled membrane receptor (TGR5) and the nuclear farnesoid X receptor (FXR), with both receptors being expressed in multiple organs, such as the liver, renal system, brain, heart, and retinas.^9^ In addition to the regulation of metabolic homeostasis, bile acids and their receptors have been implicated in the regulation of the inflammatory response.^11^, ^12^, ^13^ It has been reported that TGR5 prevents TNF‐α‐induced adhesion molecule expression.^14^ Moreover, bile acids also activate ion channels, including the bile acid‐sensitive ion channel and epithelial Na^+^ channel. Whether bile acids modulate neuropathic pain by inhibiting neuroinflammation in the spinal dorsal horn is still unknown.

Imbalance of the excitatory–inhibitory transmission contributes to neuropathic pain pathogenesis.^15^ Peripheral nerve injury promotes a loss of inhibitory transmission in the spinal cord dorsal horn, and GABA_A_ receptor‐mediated phasic or tonic currents were shown to be decreased in a neuropathic pain model, chronic constriction of the sciatic nerve (CCI).^16^ The subunit composition of GABA_A_ receptors at synapses differs from that of extrasynaptic receptors, and these two types of GABA_A_ receptors mediate two different forms of inhibition: synaptic and tonic inhibition.^17^ Activation of the GABA_A_ receptors located perisynaptically or extrasynaptically by ambient GABA is constantly present in the extracellular space, generating a constant current flow through the membrane that underlies tonic inhibition.^18^ GABA_A_ receptors that contain the δ subunit are restricted to extrasynaptic locations,^19^ making them likely mediators of tonic inhibition.^20^, ^21^

The tonic inhibitory currents mediated by extrasynaptic GABA_A_ receptors modulate neuronal excitability and synaptic transmission. Neurosteroids potentiate GABA_A_ receptors' function. It has been suggested that TGR5 acts as a neurosteroid receptor.^22^ Bile acids, which are natural products derived from cholesterol, can modulate the function of neurotransmitter receptors, such as the muscarinic acetylcholine receptors and GABA_A_ receptors, NMDA receptors and P2X2 receptor,^23^, ^24^ revealing their neuroactive potential activity. Therefore, we hypothesized that bile acids play a critical role in inhibiting neuropathic pain by modulating the functional properties of GABA_A_ receptors following peripheral nerve injury.

Therefore, we hypothesized that the activation of bile acid receptors could inhibit neuroinflammatory responses in the spinal cord by modulating GABA_A_ receptor and thereby alleviate neuropathic pain. Here, we studied the changes in total bile acid levels, the alterations in bile acid rate‐limiting synthesis enzymes, and bile acid receptor expression in the lumbar spinal dorsal horn after peripheral nerve injury. The effects and mechanisms by which exogenous bile acid receptor agonists inhibit neuropathic pain were also investigated.

## 2 MATERIALS AND METHODS

This study was not preregistered.

### 2.1 Animals

Male C57BL/6 mice weighing 25–31 g were obtained from the Animal Experimental Center, Sun Yat‐sen University, China. Mice were housed with free access to sterile water and standard laboratory food in a temperature‐controlled room maintained at 24°C ± 1°C and 50%–60% humidity. All experimental procedures were approved by the Animal Care Committee of Sun Yat‐sen University (No. SYXK (yue) 2017–0081) and were carried out following the Regulations for the Administration of Affairs Concerning Experimental Animals (China) and the ethical guidelines for the investigation of experimental pain in conscious animals (Zimmermann 1983). All of the experiments were conducted during the light phase in a double‐blinded manner. No statistical methods were used to predetermine the sample size but were based on our previous experience.^25^, ^26^

**FIGURE 1:**
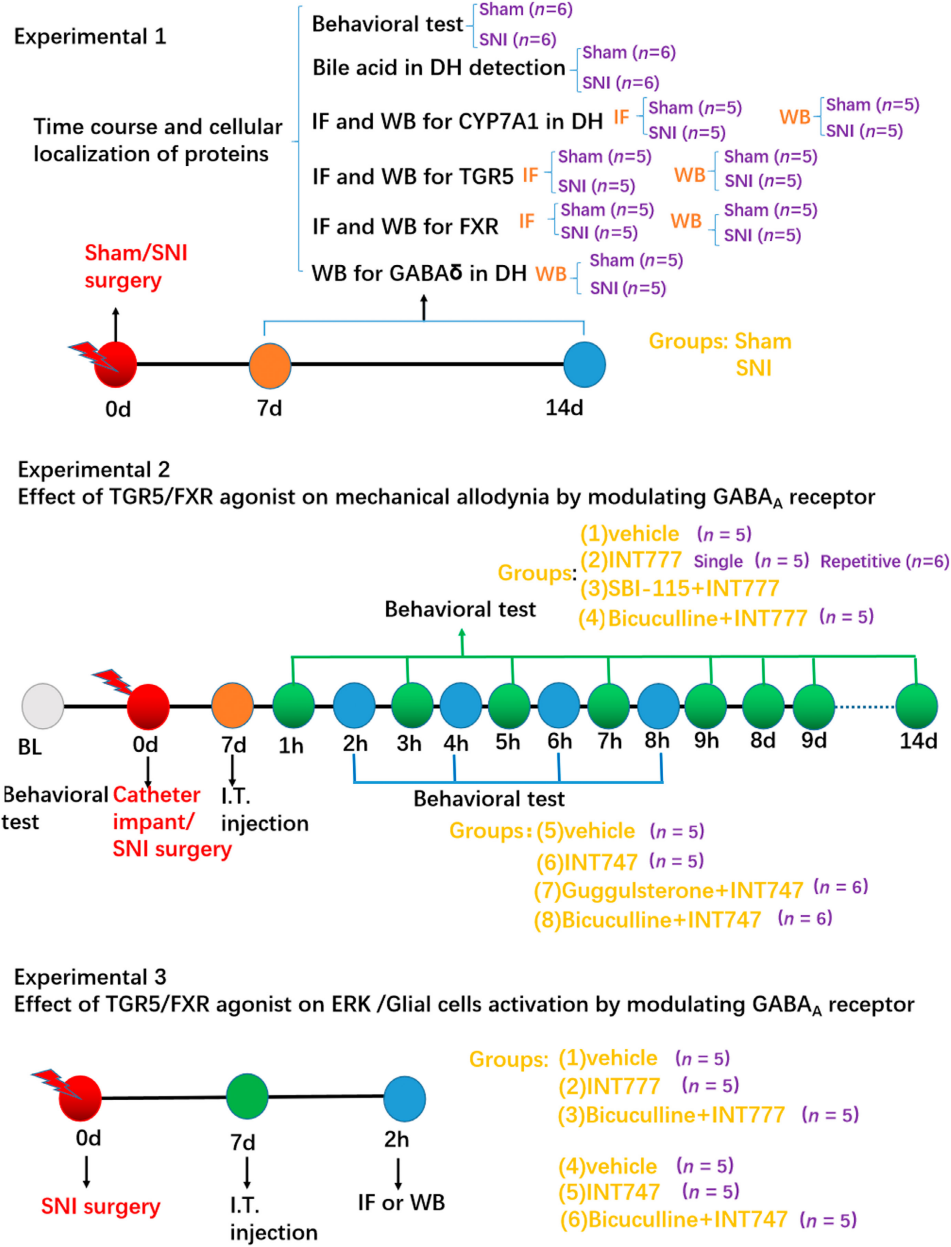
Experimental design and animal group classification. In experiment 1, after the baseline 50% paw withdrawal threshold (50% PWT) detection on day −3, mice received SNI surgery on day 0, after which the changes in bile acids and expression of CYP7A1, and TGR5/FXR in the spinal dorsal horn were investigated by western blotting and immunofluorescence on days 7 and 14 after the surgery. In experiment 2, an intrathecal catheter was implanted, and SNI surgery was performed on day 0. After recovery for 7 days, TGR5 agonist INT‐777 or FXR agonist INT‐747 was injected into the subarachnoid space via the catheter, the 50% PWT was measured by behavioral test at 1–9 h and 1–6 days after the drug treatment. SBI‐115, Z‐guggulsterone or bicuculline was injected intrathecally to block TGR5, FXR, or GABA_A_ receptor, respectively. In experiment 3, the molecular changes including p‐ERK, Iba1, and GFAP in the spinal cord at 2 h after the intrathecal injection of INT‐777 or INT‐747 were investigated by western blotting and immunofluorescence, whether the changes were abolished by GABA_A_ antagonist was also investigated. BL, baseline; IF, immunofluorescence; SNI, spared nerve injury; WB, western blotting.

In total, 177 mice were used in the present study; among them, two mice died after surgery and one mouse was euthanized due to excessive suffering from pain. Eight mice were excluded because no mechanical allodynia was induced after SNI surgery or because intrathecal catheterization failed. The experimental design and animal group classfication are shown in Figure 1.

### 2.2 Assessment of mechanical sensitivity

The mechanical sensitivity of mice was assessed with the up–down method following a previous study^25^, ^27^ using a series of filaments with logarithmically incremental stiffness (0.4, 0.6, 1, 1.4, 2, 4, 6, and 8) (Stoelting Co). After habituation on a Plexiglass box with a wire grid floor, filaments were applied in either ascending or descending strengths to determine the filament strength closest to the hind paw withdrawal threshold. Each filament was applied for a maximum of 6 s in each trial. Quick withdrawal or licking the paw in response to the stimulus was considered a positive response. The 50% paw withdrawal threshold was then calculated according to a previous study.^27^

### 2.3 Western blotting

After a defined survival time, the lumbar spinal dorsal horn was dissected and homogenized in 15 mM Tris buffer (pH 7.6; 250 mM sucrose, 1 mM magnesium chloride, 1 mM dithiothreitol, 2.5 mM EDTA, 1 mM EGTA, 50 mM sodium fluoride, 10 μg/mL leupeptin, 1.25 μg/mL pepstatin, 2.5 μg/mL aprotin, 2 mM sodium pyrophosphate, 0.1 mM sodium orthovanadate, 0.5 mM phenylmethylsulfonyl fluoride, and protease inhibitor mixture; Roche Molecular Biochemicals). The samples were sonicated and then centrifuged at 13,000 × g for 20 min to isolate the supernatant‐containing protein samples. Proteins were separated by sodium dodecyl sulfate–polyacrylamide gel electrophoresis (SDS–PAGE) and transferred onto a polyvinylidene fluoride membrane (Bio‐Rad Laboratories, Inc.). The blots were incubated in blocking buffer [5% nonfat milk dissolved in Tris‐buffered saline (TBS) containing 50 mM Tris–HCl (pH 7.6) and 150 mM NaCl] for 1 h at room temperature, followed by incubation with primary antibodies against TGR5 (1:1000, Novus), FXR (1:1000, Byorbyt), CYP7A1 (1:1000, Millipore), ERK (1:1000, Cell Signaling Technology), and p‐ERK (1:1000, Cell Signaling), overnight at 4°C. The membranes were washed with TBS‐Tween‐20 three times and incubated with horseradish peroxidase‐conjugated rabbit anti‐goat, goat anti‐mouse, or goat anti‐rabbit IgG secondary antibodies for 1 h at room temperature. The blots were developed with enhanced chemiluminescence (Clarity™ Western ECL Substrate, Bio‐Rad) detected by a Tanon 5200 imager (Tanon Science & Technology Co., Ltd.), and analyzed by Tanon MP software (Tanon Science & Technology Co., Ltd.). The intensities of the blots were quantified and normalized against a loading control (β‐actin) by an investigator blinded to the experimental conditions.

### 2.4 Immunofluorescence staining

Mice were anesthetized with pentobarbital and transcardially perfused with 0.9% saline followed by 4% paraformaldehyde in 0.1 M phosphate buffer (PB). The lumbar spinal cords were dissected, postfixed for 3 h, and kept overnight in 30% sucrose in 0.1 M PB. The spinal cords (25 μm sections) were cut on a cryostat (Leica CM 1900). For immunofluorescence staining, lumbar spinal cord sections were first blocked with 1% donkey serum in 0.3% Triton for 1 h at room temperature and then incubated overnight at 4°C with a primary antibody for TGR5 (1:200, Novus), FXR (1:200, Byorbyt), CYP7A1 (1:200, Millipore), glial fibrillary acidic protein (GFAP, 1:500, Cell Signaling Technology), neuronal‐specific nuclear protein (NeuN) (1:500, mouse monoclonal; Chemicon), or goat polyclonal anti‐ionized calcium‐binding adaptor molecule 1 (Iba1, 1:500, Abcam). The sections were then incubated for 1 h at room temperature with a corresponding secondary antibody (1:300; Invitrogen).

For double staining, secondary primary antibodies were added after incubation with the primary antibodies for the same procedure as described above.

For quantification of immunofluorescence staining, the optical density of positive signals per section was measured by ImageJ Version: k 1.45. Every fifth section was picked from a series of sections, 4–6 sections for each animal were selected randomly, and the average was calculated.

### 2.5 Intrathecal injection

For single intrathecal injection, direct transcutaneous intrathecal injection was performed following previous work.^28^, ^29^ Briefly, a 25‐Ga 3 10 needle connected to a Hamilton syringe was inserted into the tissues between the dorsal aspects of L5 and L6 of mice, perpendicular to the vertebral column. The injection was considered successful if a tail flick was observed immediately. Then, 5 μL INT‐777, INT‐747, bicuculline SBI‐115, or Z‐guggulsterone (all the above drugs were purchased from MedChemExpress) was administered. For repetitive INT‐777 injection, the drug was injected intrathecally via a polyethylene 10 (PE‐10) tube, the tip of which was placed on the spinal lumbar enlargement level 1 week before. Whether the tube was correctly placed in the intervertebral space was confirmed by paralysis in the bilateral hind limb after a 2% lidocaine injection (3 μL) through the catheter within 30 s.

### 2.6 Bile acid quantification

The lumbar spinal cord tissue was dissected under 0.5% pentobarbital sodium (50 mg/kg) anesthesia, after which approximately 10–25 mg of the tissue was homogenized in 1 mL of a dry‐ice chilled solution containing 80% methanol in H_2_O. The homogenates were then centrifuged at 13,000 *g* for 15 min to remove the insoluble fraction. The supernatants that contained soluble metabolites were then lyophilized and subsequently resuspended in 50 μL of sterile H_2_O. The amount of bile acids was detected using a bile acid assay kit (Sigma–Aldrich), which provided a convenient fluorimetric means to measure total bile acid, according to the manufacturer's guidelines. 3α‐Hydroxysteroid dehydrogenase reacts with bile acid, converting NAD to NADH and reducing a probe to a highly fluorescent product. The profiles of bile acids were determined by a fluorescence microplate reader (λex = 530 nm/λem = 585 nm). The results were then normalized by the tissue weight.

### 2.7 Statistical analysis

Data are expressed as the mean ± SEM and were analyzed with GraphPad Prism 8.0 Software. Shapiro–Wilk tests for normality were used to assess data distribution. Data were considered non‐normally distributed if *p* < 0.05. Data that do not exhibit a normal distribution were analyzed via non‐parametric tests, including the Friedman ANOVA for repeated measurements, followed by the Wilcoxon matched‐pairs test or the Mann–Whitney U test. The normally distributed data were analyzed by one‐way ANOVA followed by Tukey's post hoc test or two‐tailed unpaired Student's *t*‐test. *p* < 0.05 was considered statistically significant. When non‐parametric tests are used, they are specified in the figure legend. No statistical methods were used to predetermine the sample size, which was based on our previous experience. No tests for outliers were conducted on the data.

## 3 RESULTS

### 3.1 SNI induced mechanical allodynia, decreased the level of bile acids and upregulated CYP7A1 in microglia in the lumbar spinal dorsal horn

**FIGURE 2:**
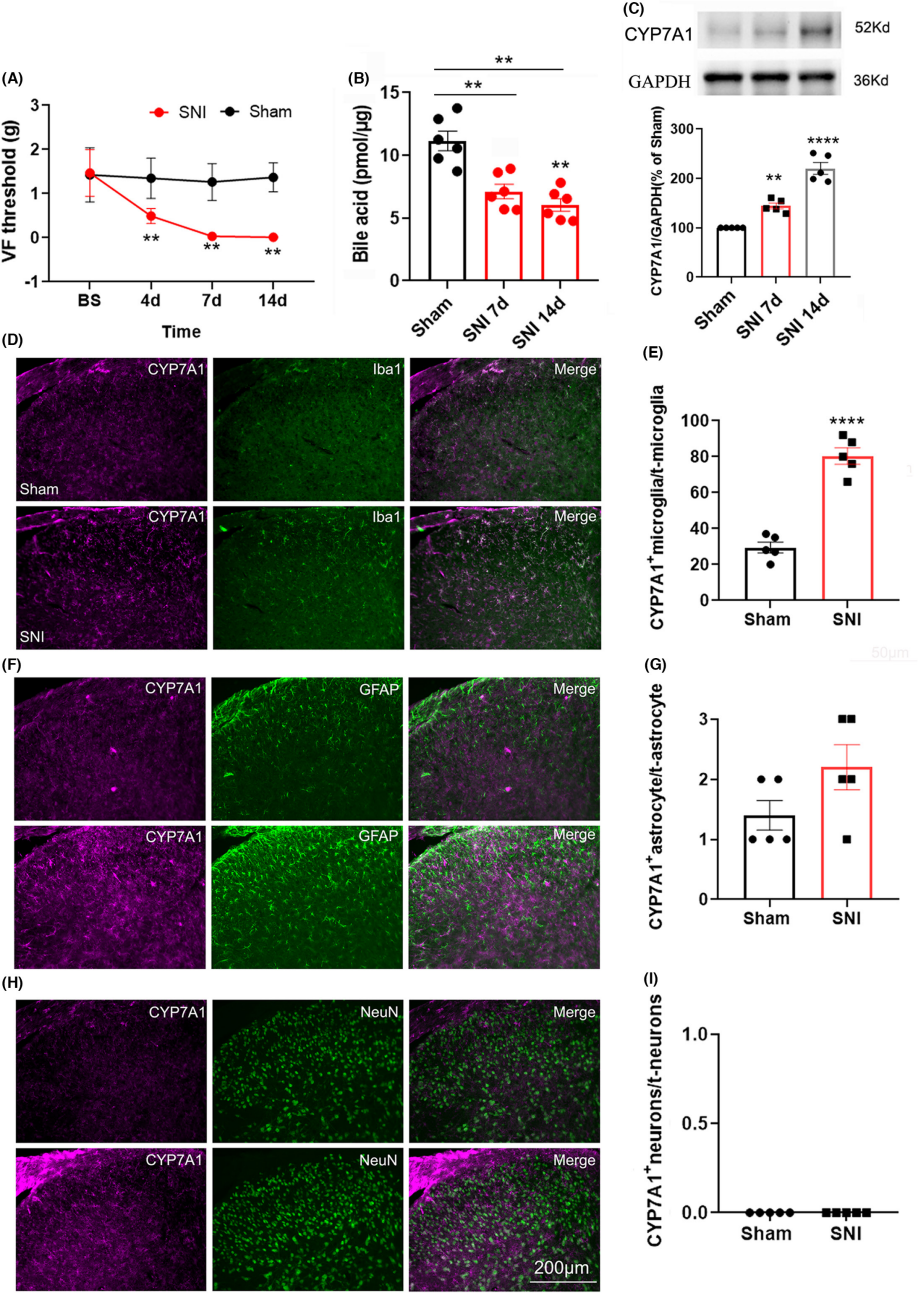
SNI decreased the 50% paw withdrawal threshold, decreased the level of total bile acids, and upregulated CYP7A1 in microglia in the lumbar spinal dorsal horn. (A) SNI decreased the 50% paw withdrawal threshold on the ipsilateral hind paw on days 4, 7, and 14. **p < 0.01 compared with the sham group (Mann–Whitney U test, *n* = 6/group). (B) The level of total bile acids in the lumbar spinal dorsal horn on day 7 and day 14 after SNI. **p < 0.01 compared with the sham group. (C) Western blotting shows the expression of CYP7A1 in the ipsilateral lumbar spinal dorsal horn 7 and 14 days after SNI or sham operation. The quantification of CYP7A1 normalized to β‐actin is shown under the representative bands (*n* = 5/group). **p < 0.01 compared with the sham group. (D, F, and H) In the ipsilateral lumbar spinal dorsal horn, CYP7A1 was located mainly in Iba1‐labeled microglia (D) and to a much lesser extent in GFAP‐labeled astrocytes (F) but not in NeuN‐marked neurons (H) 7 days after either sham or SNI operation. (F, G and I), The percentage of microglia, astrocytes and neurons that expressed CYP7A1 in sham and SNI mice (day 7). Bar = 200 μm. ****p < 0.0001 compared with the sham group.

Consistent with previous reports, SNI decreased the 50% paw withdrawal threshold on day 4 and day 7, which lasted until day 14 (Figure 2A). Furthermore, we found that the level of total bile acids in the lumbar spinal dorsal horn was significantly decreased on days 7 and 14 after SNI (Figure 2B). To test whether SNI changed the expression of cholesterol 7 α‐hydroxylase (CYP7A1), a rate‐limiting enzyme for bile acid synthesis, in the spinal dorsal horn, we performed western blotting and immunofluorescence staining. The western blotting results showed that compared with the sham group (Figure 2C), the expression of CYP7A1 protein in the ipsilateral lumbar spinal dorsal horn was increased significantly on days 7 and 14 after SNI. Immunohistochemistry further confirmed the western blotting results (Figure 2D–I). To determine the types of cells in which CYP7A1 was expressed, double immunofluorescence staining of CYP7A1 with three cell‐specific markers, NeuN (a marker for neurons), GFAP (a marker for astrocytes), and Iba1 (a marker for microglia), was performed on lumbar spinal cord sections from sham‐operated and SNI mice. The results showed that CYP7A1 was located mainly in microglia (Figure 2D) and to a much lesser extent in astrocytes (Figure 2F) but not in neurons (Figure 2H) in either sham or SNI mice. Compared with the sham group, the percentages of microglia (Figure 2E) that expressed CYP7A1‐IR increased, while the percentage of astrocytes (Figure 2G) and neurons (Figure 2I) that expressed CYP7A1‐IR did not change significantly on day 7 after SNI. These results indicated that the synthesis of bile acids in microglia of the spinal dorsal horn was increased after peripheral nerve injury.

### 3.2 TGR5 was upregulated in the spinal dorsal horn by SNI

**FIGURE 3:**
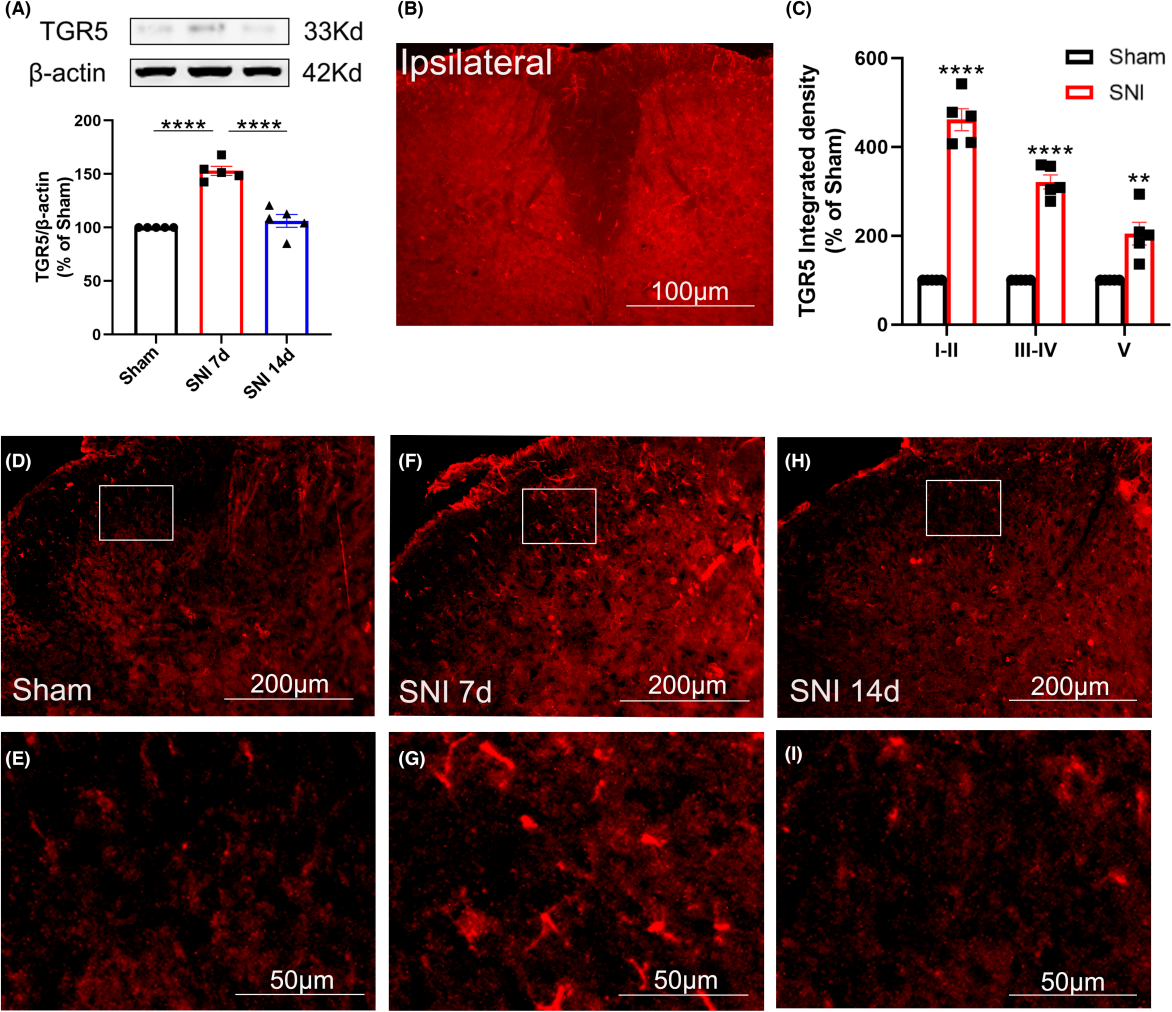
TGR5 was upregulated in the spinal dorsal horn after SNI. (A) Western blotting shows the expression of TGR5 in the ipsilateral lumbar spinal dorsal horn 7 and 14 days after SNI or sham operation. The quantification of TGR5 normalized to β‐actin is shown under the representative bands (*n* = 5/group). (B) Changes in TGR5‐IR in the bilateral lumbar spinal dorsal horn on day 7 after SNI. (C) Quantification of TGR5‐IR optic density in different laminas in the ipsilateral lumbar spinal dorsal horn on day 7 after sham or SNI surgery (*n* = 5/group). **p < 0.01, ****p < 0.0001 compared with the sham group. (D–H) Representative experiments show the change in TGR5‐IR in the ipsilateral lumbar spinal dorsal horn of sham and SNI mice (days 7 and 14). (E–I) Magnified picture of the boxed area in D‐H, respectively (*n* = 5/group).

**FIGURE 4:**
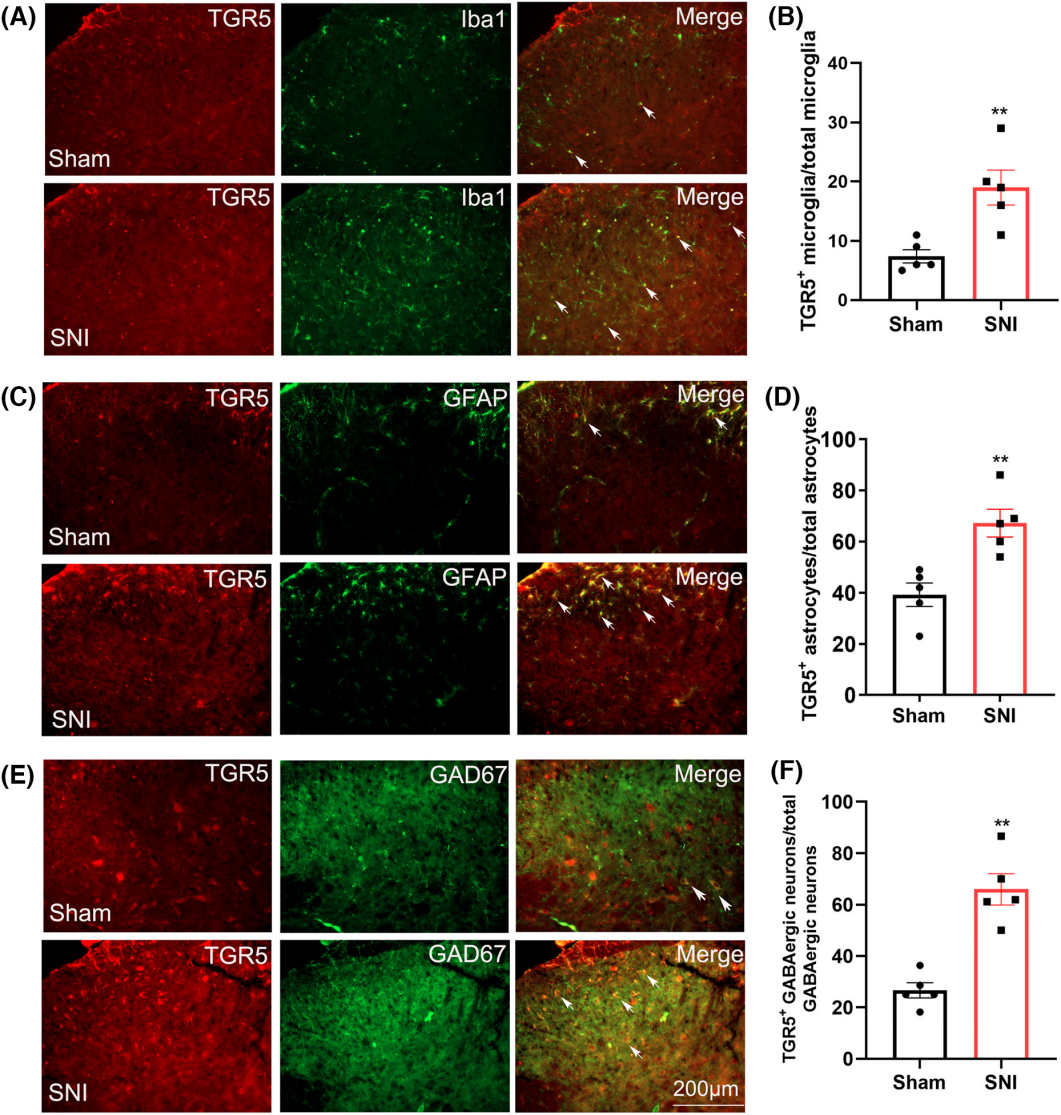
The cell types that expressed TGR5 in the spinal dorsal horn of sham and SNI mice. (A, C, and E) In the ipsilateral lumbar spinal dorsal horn, TGR5 was located in Iba1‐labeled microglia (A), GFAP‐labeled astrocytes (C), and GAD67‐marked inhibitory neurons (E) 7 days after the sham and SNI operations. (B, D, and F) The percentage of microglia, astrocytes, and GAD67‐positive neurons that expressed TGR5 in sham and SNI mice (day 7). Bar = 200 μm.

We next tested whether the expression of the bile acid membrane receptor TGR5 in the spinal dorsal horn was changed after SNI. The results showed that 7 days after SNI, the protein expression of TGR5 was significantly upregulated and returned to the baseline level on day 14 (Figure 3A). Immunofluorescence experiments further confirmed the western blotting data (Figure 3B–I), showing that on day 7 after SNI, TGR5‐IR was located in the bilateral spinal dorsal horn (Figure 3B) although SNI surgery was performed unilaterally at the left hind paw. Compared with the sham group, the increase in TGR5‐IR was mainly located in laminae I–II, to a lesser extent in laminae III–IV, and much less in laminae V on day 7 after SNI (Figure 3C). Double immunofluorescence staining further showed that on day 7, TGR5 was located in Iba1‐positive microglia (Figure 4A), GFAP‐positive astrocytes (Figure 4C), and GAD67‐positive neurons (Figure 4E) in both sham and SNI mice. Compared to the sham group, each percentage of microglia (Figure 4B), astrocytes (Figure 4D), and GAD67‐positive neurons (Figure 4F) that expressed TGR5 was increased on day 7 after SNI, suggesting that the expression of TGR5 is widely distributed.

### 3.3 Activation of TGR5 alleviated the established neuropathic pain induced by SNI, and the effect was blocked by SBI‐115 or bicuculline

**FIGURE 5:**
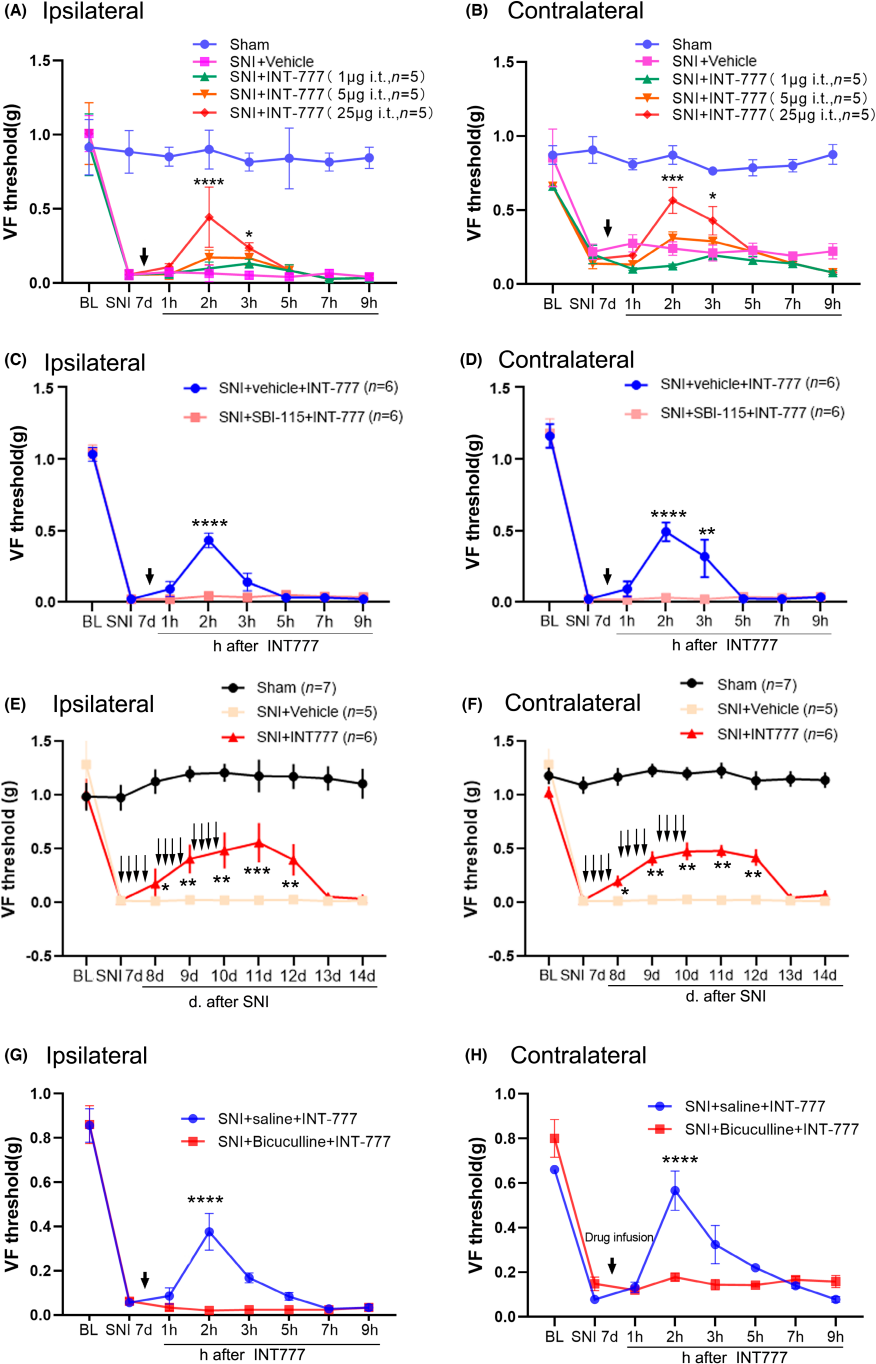
Intrathecal injection of the TGR5 agonist INT‐777 alleviated the mechanical allodynia in SNI mice, and the effect was prevented by TGR5 or GABA_A_ antagonist. (A, B) A single intrathecal injection of INT‐777 on day 7 after SNI dose dependently attenuated the mechanical allodynia in bilateral hind paw (*n* = 5 or 6/group). (C, D) The effect of single INT‐777 on the 50% paw withdrawal threshold was blocked by pretreatment with TGR5 antagonist SBI‐115 (*n* = 6/group). (E, F) Repetitive injection of INT‐777, starting on day 7 after SNI and then four times daily for a total of 3 days significantly increased the 50% paw withdrawal threshold 1–5 days after drug application (*n* = 5‐7/group). (G, H), The effect of single INT‐777 on the 50% paw withdrawal threshold was blocked by GABA_A_ receptor antagonist bicuculline (*n* = 6/group). The downward arrows indicate the drug injection. *p < 0.05, **p < 0.01, ***p < 0.001, and ****p < 0.0001 compared with SNI day 7, Friedman ANOVA for repeated measurements, followed by the Wilcoxon matched‐pairs test.

Having demonstrated that the concentration of bile acids and the expression of its receptor TGR5 in the spinal dorsal horn were changed after peripheral nerve injury. We next tested whether activation of TGR5 could affect neuropathic pain behaviors. To do so, a single intrathecal injection of the TGR5 agonist INT‐777 was performed on day 7 after SNI, and the 50% paw withdrawal threshold (50% PWT) was tested before and after the treatment. The results showed that INT‐777 suppressed the established mechanical allodynia in bilateral hind paw in a dose‐dependent manner (Figure 5A,B). INT‐777 at 1 and 5 μg in 5 μL had no obvious effects on the 50% PWT, whereas 25 μg INT‐777 significantly increased the 50% PWT in the ipsilateral and contralateral hind paw 2 and 3 h after the drug treatment (compared to the predrug, SNI day 7, Figure 5A,B). INT‐777 injection had no obvious effect on the 50% PWT of the sham mice. The effect of single INT‐777 on 50% PWT in SNI mice was completely abolished by intrathecal injection of TGR5 antagonist SBI‐115 (15 μg in 5 μL) (Figure 5C,D). To test whether INT‐777 had a longer‐term effect, INT‐777 (25 μg, 5 μL) was repetitively injected intrathecally via a PE‐10 tube, starting on day 7 after SNI and then four times daily, for a total of 3 days. To distinguish the lasting effect from the acute effect, the 50% PWT was detected at least 4 h after the last INT‐777 application. The results showed that a long‐lasting increase in the 50% PWT was observed (Figure 5E,F).

The tonic inhibitory currents mediated by extrasynaptic GABA_A_ receptors modulate neuronal excitability and synaptic transmission. To determine whether SNI attenuated GABA_A_ receptor δ subunit expression, one main subunit that constitutes extrasynaptic GABA_A_ receptors,^19^ we conducted western blotting experiments, which confirmed that there was a significant decrease in GABA_A_ receptor δ subunit expression in SNI mice on day 7 compared with sham controls (Figure S1). These results suggest that GABA_A_ receptor‐mediated tonic inhibition declined after SNI.

Bile acids can modulate the function of neurotransmitter receptors, including GABA_A_ receptors.^23^, ^24^ Therefore, we next tested whether bile acids played a critical role in neuropathic pain by modulating the functional properties of GABA_A_ receptor. The results demonstrated that intrathecal injection of the GABA_A_ receptor antagonist bicuculline (125 μg, 5 μL), which was applied 30 min before INT‐777 injection, completely blocked the increasing effect of INT‐777 on the 50% PWT on bilateral hind paws in SNI mice (Figure 5G,H).

### 3.4 Activation of TGR5 by INT‐777 inhibited the expression of p‐ERK and the activation of glial cells in the spinal dorsal horn, and the effect was blocked by bicuculline

**FIGURE 6:**
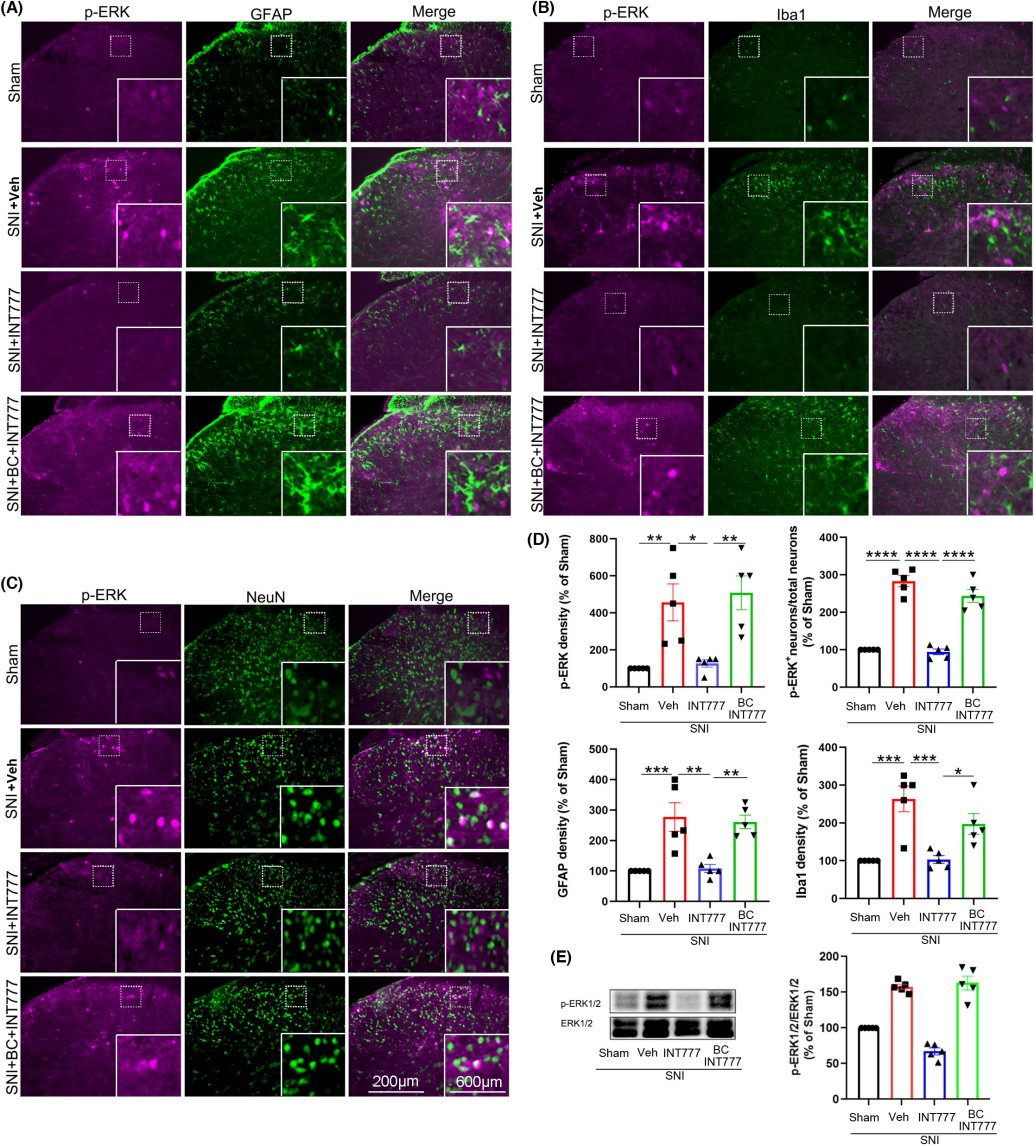
Intrathecal injection of INT‐777 inhibited ERK phosphorylation and glial cell activation in the spinal dorsal horn, and these effects were abolished by bicuculline. (A) Representative results of p‐ERK and GFAP expression in the spinal dorsal horn in the sham, SNI, INT‐777‐treated SNI, and BIC‐ and INT‐777‐treated SNI groups. (B) Representative results of p‐ERK and Iba1 expression in the spinal dorsal horn in the sham, vehicle (Veh)‐treated SNI, INT‐777‐treated SNI, and bicuculline (BC)‐treated SNI groups. (C) Representative results of p‐ERK and NeuN expression in the spinal dorsal horn in the four different groups. (D) Quantification of the density of p‐ERK, GFAP, and Iba1 and the percentage of total neurons that expressed p‐ERK in the four groups. (E) INT‐777 treatment significantly decreased the expression of p‐ERK in the spinal dorsal horn, which was significantly increased after SNI. Pretreatment with bicuculline blocked the effect of INT‐777 on p‐ERK expression. *p < 0.05, **p < 0.01, ***p < 0.001, and ****p < 0.0001 compared with the related group.

It has been well established that neuroinflammation following glial cell activation in the spinal cord is important for the maintenance of neuropathic pain.^30^, ^31^ Therefore, we further investigated whether INT‐777 inhibited the activation of glial cells and the expression of p‐ERK, which indicates sensitization of neurons and has been reported to modulate various types of pain.^7^ Immunostaining experiments showed that compared to the sham‐operated mice, the shapes of both GFAP‐positive and Iba1‐positive cells became hypertrophic with thick, short projections, and the expression of Iba1, GFAP, and p‐ERK in neurons was significantly increased on day 7 after SNI (Figure 6A–D), indicating astrocytes and microglia were activated and neurons were excited. A single intrathecal injection of INT‐777 (25 μg in 5 μL) on day 7 after SNI significantly inhibited the expression of Iba1 and GFAP and neuronal p‐ERK in the spinal dorsal horn at 2 h; moreover, the shapes and numbers of astrocytes and microglia were comparable to those from the sham group (Figure 6A–D). These results indicated that INT‐777 inhibited the activation of glial cells and neurons in SNI mice. Furthermore, bicuculline (125 μg, 5 μL), which was applied intrathecally 30 min before INT‐777 injection, blocked the inhibitory effect of INT‐777 on the activation of glial cells and the expression of p‐ERK (Figure 6A–E).

### 3.5 FXR was upregulated in the spinal dorsal horn by SNI

**FIGURE 7:**
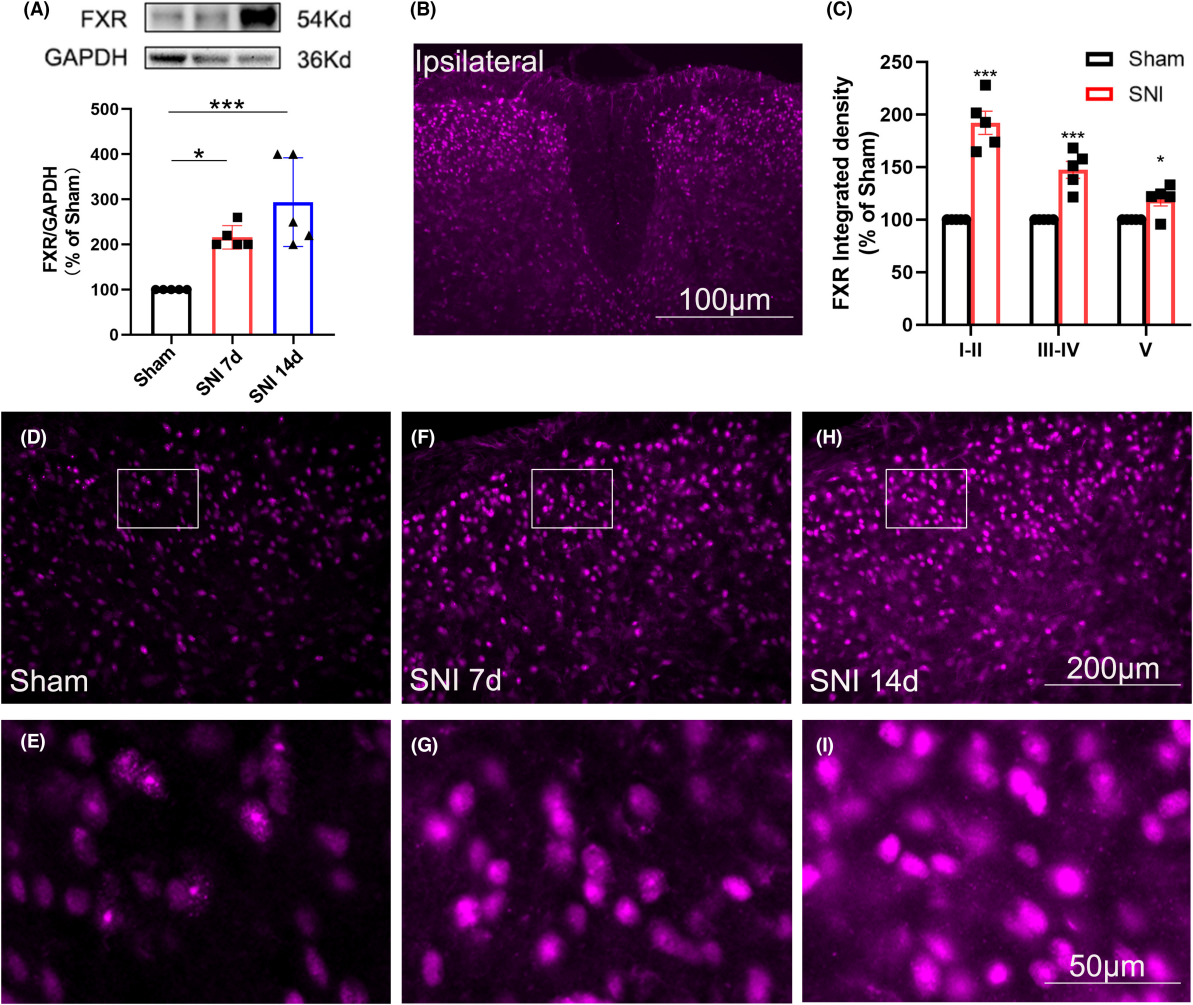
FXR was upregulated in the spinal dorsal horn after SNI. (A) Western blotting shows the expression of FXR in the ipsilateral lumbar spinal dorsal horn 7 and 14 days after SNI or sham operation. The quantification of FXR normalized to GAPDH is shown under the representative bands (*n* = 5/group). (B) Changes in FXR‐IR in the bilateral lumbar spinal dorsal horn on day 7 after SNI. (C) Quantification of FXR‐IR optic density in different laminae in the ipsilateral lumbar spinal dorsal horn 7 days after sham or SNI surgery (*n* = 5/group). **p < 0.01, ****p < 0.0001 compared with the sham group. (D–H) Representative experiments show the changes in FXR‐IR in the ipsilateral lumbar spinal dorsal horn in sham‐operated mice and mice that received SNI at different time points, as indicated. (E–I) Magnified picture of the boxed area in D–H (*n* = 5/group).

**FIGURE 8:**
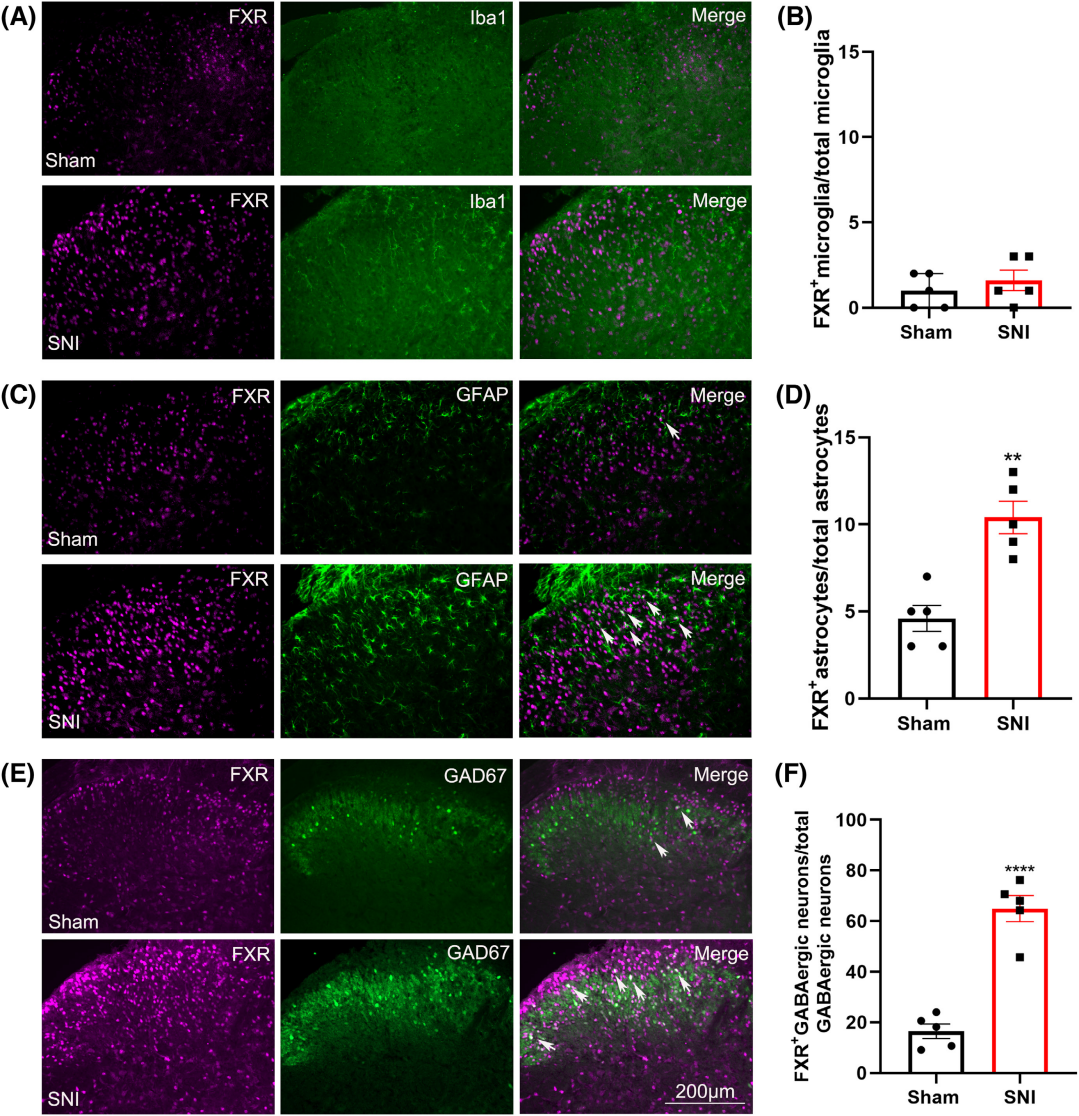
The cell types that expressed FXR in the spinal dorsal horn from sham and SNI mice. (A, C, and E) In the ipsilateral lumbar spinal dorsal horn, FXR was located in GFAP‐labeled astrocytes (C) and GAD67‐GFP‐positive neurons (E) but not in Iba1‐labeled microglia (A) 7 days after sham and SNI operations. (B, D, and F) The percentages of microglia, astrocytes, and GAD67‐GFP‐positive neurons that expressed FXR in sham and SNI mice on day 7 are shown. **p < 0.01, ****p < 0.0001 compared with the sham group. Bar = 200 μm.

We next tested whether the expression of the bile acid nuclear receptor FXR in the spinal dorsal horn was changed after SNI. The results showed that 7 days after SNI, the protein expression of FXR was significantly increased, which was further increased on day 14 (Figure 7A). Immunofluorescence experiments further confirmed the western blotting data (Figure 7B–I), showing that on day 7 after SNI, FXR‐IR was located in the bilateral spinal dorsal horn. Compared with the sham group, FXR‐IR increased mostly in laminae I–II, to a lesser extent in laminae III–IV, and much less in laminae V on day 7 after SNI (Figure 7C). Double immunofluorescence staining showed that on day 7, FXR was located mainly in glutamate decarboxylase 67 (GAD67)‐green fluorescent protein (GFP) neurons and to a lesser extent in GFAP‐positive astrocytes and Iba1‐positive microglia (Figure 8A,C,E). Except for microglia, the percentage of astrocytes and GAD67‐GFP‐positive neurons that expressed FXR was increased on day 7 after SNI compared to the sham group, suggesting that the expression of FXR increased in astrocytes and GABAergic neurons after SNI (Figure 8B,D,F).

### 3.6 Activation of FXR alleviated the established neuropathic pain induced by SNI, and the effect was blocked by guggulsterone or bicuculline

**FIGURE 9:**
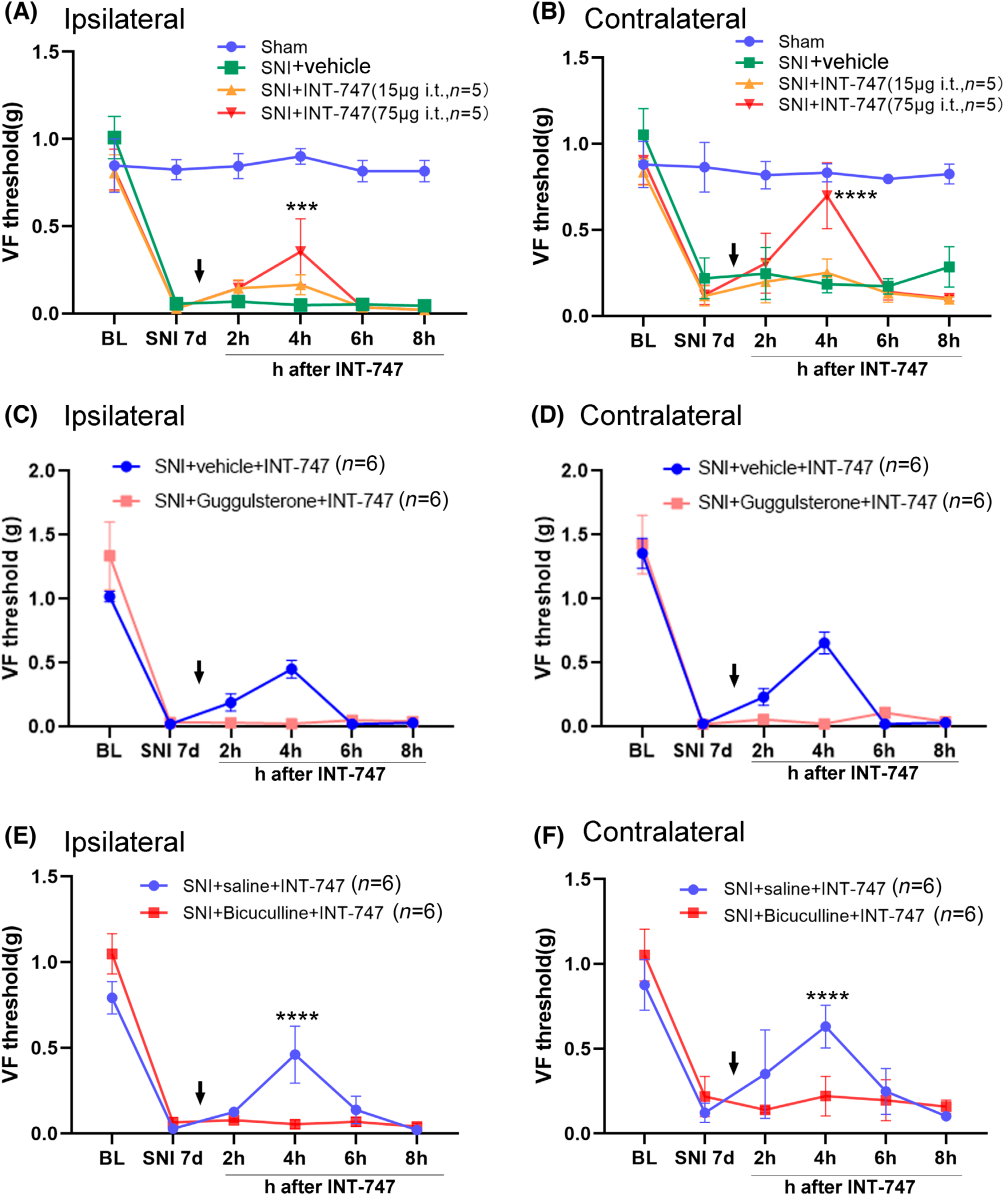
Intrathecal injection of the FXR agonist INT‐747 alleviated the mechanical allodynia in SNI mice, and the effects were blocked by FXR or GABA_A_ antagonist. (A, B) A single intrathecal injection of INT‐747 on day 7 after SNI dose dependently attenuated mechanical allodynia in the ipsilateral (A) and contralateral hind paw (B) in SNI mice (*n* = 5 or 6/group). (C, D) The effect of INT‐747 (25 μg) on the 50% paw withdrawal threshold in bilateral hind paws was blocked by FXR antagonist Z‐guggulsterone (*n* = 6/group). (E, F) The effect of INT‐747 on the 50% paw withdrawal threshold in bilateral hind paws was blocked by bicuculline (*n* = 6/group). ***p < 0.001, ****p < 0.0001 compared with SNI on day 7, Friedman ANOVA for repeated measurements, followed by the Wilcoxon matched‐pairs test.

We next tested whether activation of FXR by intrathecal injection of INT‐747 could alleviate the neuropathic pain behaviors induced by SNI. Behavioral tests showed that a single intrathecal injection of 75 μg INT‐747 on day 7 after SNI significantly increased the 50% PWT of bilateral hind paw at 4 h after drug application (Figure 9A,B). INT‐747 at 15 μg had no obvious effect on the 50% PWT (Figure 9A,B). Moreover, the increasing effect of INT‐747 on the 50% PWT was completely prevented by intrathecal injection of Z‐guggulsterone (10 μg, 5 μL) or bicuculline (125 μg, 5 μL) 30 min before INT‐747 injection (Figure 9C,F).

### 3.7 Activation of FXR by INT‐747 inhibited the expression of p‐ERK and the activation of microglia and astrocytes in the spinal dorsal horn, and the effect was blocked by bicuculline

**FIGURE 10:**
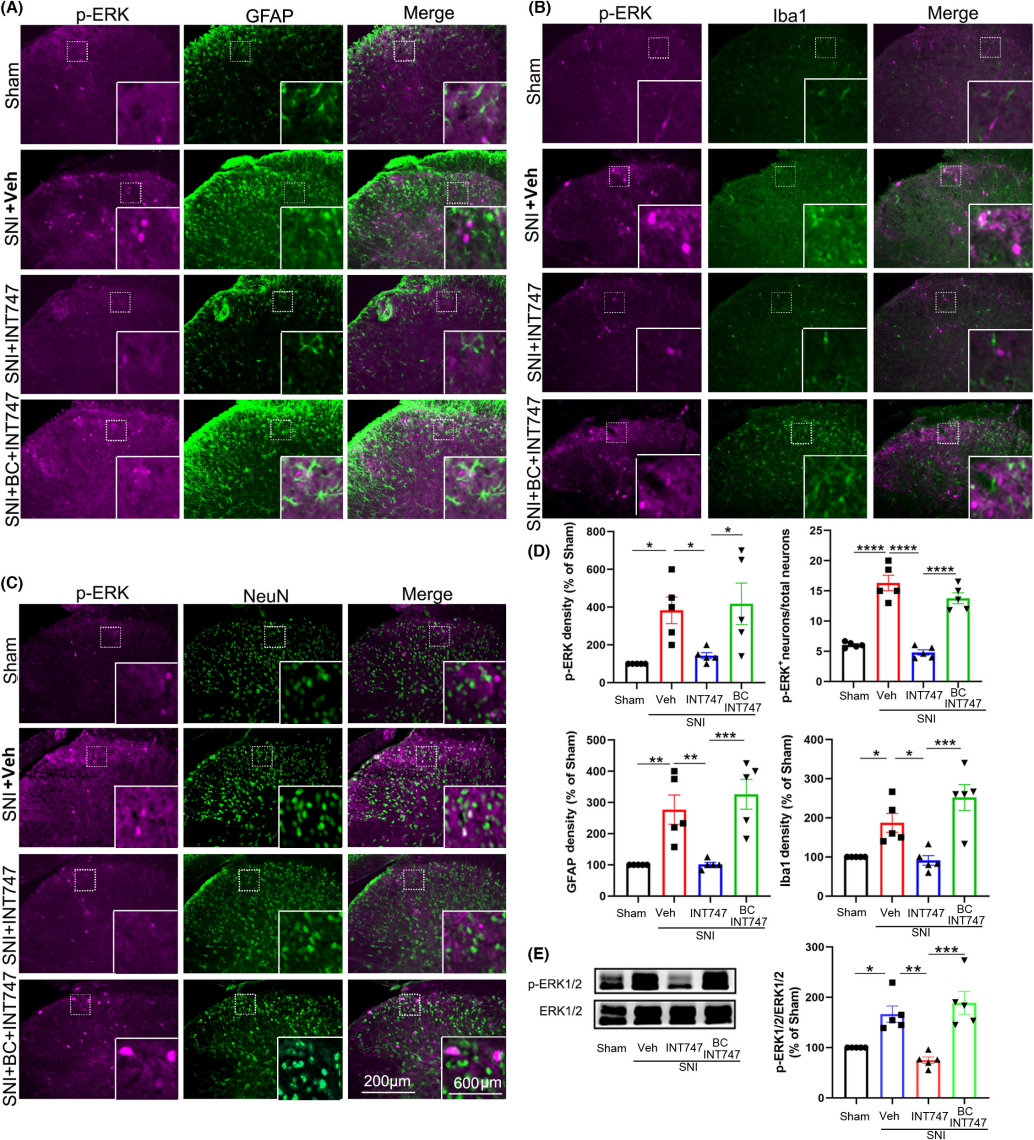
Intrathecal injection of INT‐747 inhibited ERK phosphorylation and glial cell activation in the spinal dorsal horn, and the effects were blocked by bicuculline. (A) Representative results of p‐ERK and GFAP expression in the spinal dorsal horn in sham, vehicle‐treated SNI, INT‐747‐treated SNI, and bicuculline (BC)‐ and INT‐747‐treated SNI mice. (B) Representative results of p‐ERK and Iba1 expression in the spinal dorsal horn in the sham, vehicle‐treated SNI, INT‐747‐treated SNI, and bicuculline‐ and INT‐747‐treated SNI groups. (C) Representative results of p‐ERK and NeuN expression in the spinal dorsal horn in the four different groups. (D) Quantification of the density of p‐ERK, GFAP, and Iba1 in the four different groups, as indicated. The percentage of total neurons that expressed p‐ERK in the four groups is also shown. (E) Western blot analysis showing that INT‐747 treatment significantly decreased the enhanced expression level of p‐ERK in the spinal dorsal horn in SNI mice. Pretreatment with bicuculline 30 min before INT‐747 treatment blocked its effect on p‐ERK. *p < 0.05, **p < 0.01, ***p < 0.001, and ****p < 0.0001 compared with the related group.

We further investigated whether INT‐747 inhibited the activation of glial cells and the expression of p‐ERK. Similar to INT‐777, a single intrathecal injection of INT‐747 (75 μg in 5 μL) 7 days after SNI significantly attenuated the expression of Iba1‐IR and GFAP‐IR and neuronal p‐ERK in the spinal dorsal horn at 2 h (Figure 10A,B). Moreover, mice treated with INT‐747 also showed decreased activation of glial cells and phosphorylation of p‐ERK (Figure 10A–D). Furthermore, intrathecal injection of bicuculline (125 μg, 5 μL), which was applied 30 min before INT‐747 injection, also blocked the inhibitory effect of INT‐747 on the activation of glial cells and phosphorylation of ERK (Figure 10A–E).

## 4 DISCUSSION

**FIGURE 11:**
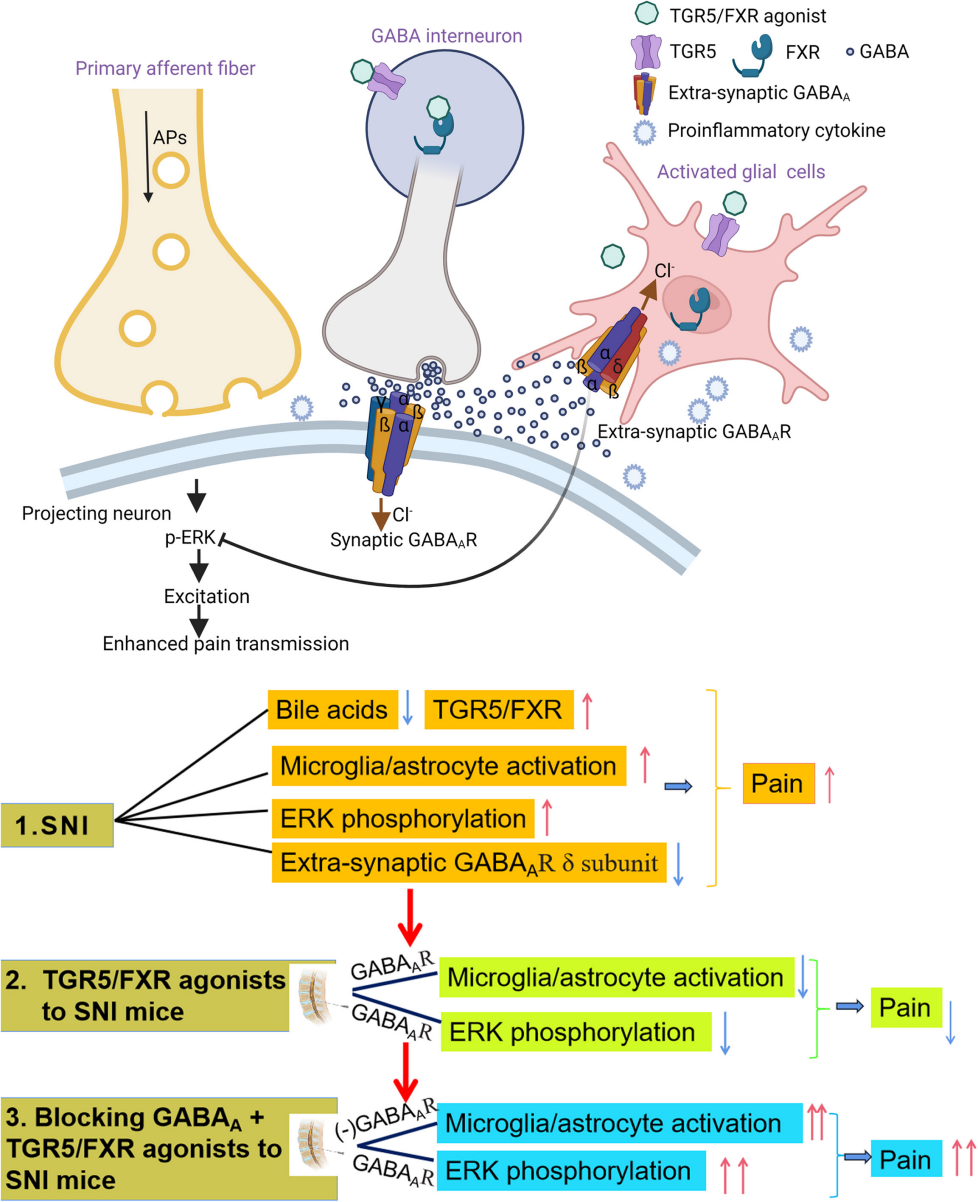
Schematic diagram depicting the role of bile acids in SNI‐induced neuropathic pain. (1) SNI induced multiple changes in the spinal dorsal horn, including decreased levels of bile acids, upregulated expression of TGR5/FXR, activated microglia/astrocytes and phosphorylated ERK in neurons, and downregulated expression of GABA_A_ receptor δ subunit. All these changes contributed to the maintenance of neuropathic pain. (2) Intrathecal injection of TGR5 or an FXR agonist alleviated neuropathic pain (pain relieved) by decreasing microglial and astrocyte activation and ERK phosphorylation in the spinal dorsal horn. (3) Effect of TGR5 or the FXR agonist on inhibition of glial cells and ERK pathway was abolished by bicuculline. Therefore, pain was aggravated.

Treatment of neuropathic pain remains a challenging problem. Here, we showed that (1) peripheral nerve injury decreased the level of bile acids, upregulated the bile acid synthesis enzyme and bile acid receptor TGR5/FXR, activated microglia/astrocytes and phosphorylated ERK in neurons, and downregulated GABA_A_ receptor δ subunit expression. All these changes contributed to the maintenance of neuropathic pain. (2) Intrathecal injection of TGR5 or an FXR agonist alleviated neuropathic pain (pain relieved) by decreasing microglia and astrocyte activation and ERK phosphorylation in the spinal dorsal horn. (3) Pretreatment with the GABA_A_ receptors' antagonist bicuculline blocked the effect of TGR5 or FXR agonists on neuropathic pain (pain aggravated) by activating glial cells and activating the ERK pathway (Figure 11). Taken together, our findings suggest that activation of the bile acid receptor in the spinal cord alleviates neuropathic pain by inhibiting the activation of glial cells, possibly by modulating function of GABA_A_ receptors, which is important for inhibitory effects in pain transduction.

### 4.1 Activation of bile acid receptors protects against neuropathic pain after peripheral nerve injury

Our previous studies have shown that the expression of liver X receptor (LXR) and translocator protein (18 kDa) (TSPO) was increased in the spinal dorsal horn after peripheral nerve injury and that either LXR agonists or TSPO agonists could alleviate neuropathic pain behaviors.^25^, ^26^ Similarly, here we showed that TGR5 and FXR were upregulated in the bilateral spinal dorsal horn after SNI and that a single intrathecal injection of their agonists (INT‐777 or INT‐747) inhibited the mechanical allodynia induced by SNI. Moreover, we demonstrated that the level of bile acids decreased, whereas the rate‐limiting enzymes of bile acid synthesis and bile acid receptors were increased in the spinal dorsal horn after SNI. Furthermore, activation of bile acid receptors alleviated mechanical allodynia induced by SNI. These findings suggest that after peripheral nerve injury, insufficient bile acid levels in the spinal cord make the mice more susceptible to neuropathic pain. Accordingly, bile acid UDCA or tauroursodeoxycholic acid (TUDCA) has neuroprotective action in several neurological diseases, including Alzheimer's disease, Parkinson's disease, Huntington's disease, multiple sclerosis, and diabetic retinopathy.^32^, ^33^, ^34^ Moreover, bear bile has been used in traditional Chinese medicine for thousands of years due to its therapeutic potential and clinical applications. This is also in line with previous reports that the TGR5 receptor mediates bile acid‐induced analgesia.^35^ Even though bile acid exerts a physiological analgesic effect, this restorative effect of endogenous bile acid biosynthesis appears insufficient to restore normal homeostasis after peripheral nerve injury; thus, exogenous supplementation of bile acid receptor agonists may be an effective strategy to terminate the state of chronic pain.

It is well known that neuropathic pain is maintained in part by central sensitization, a phenomenon of synaptic plasticity, and increased responsiveness of pain‐transmitting neurons in the spinal dorsal horn.^36^, ^37^ Accumulating evidence suggests that central sensitization is driven by neuroinflammation initiated by activated glial cells, including microglia and astrocytes in the spinal cord, which display immunological responses by releasing proinflammatory cytokines and chemokines, by antigen presentation and clearing cellular debris through phagocytosis.^38^, ^39^ Therefore, pain‐processing neurons can be sensitized by the proinflammatory mediators that are released by glial cells. From this point of view, activation of glial cells is considered detrimental to neurons and contributes to the development and maintenance of neuropathic pain due to the gain of their inflammatory function. Neuroinflammation can also exert neuroprotective effects via phagocytosis of dead neurons and clearance of debris. Glial cells can also exert intrinsic neuroprotective function by inhibiting neuroinflammation. For example, the upregulated CYP7A1 reported here, and the upregulated LXR and TSPO^25^, ^26^ that have been reported by our previous studies, are all neuroprotective molecules located in glial cells that counteract neuropathic pain.

The mechanism by which CYP7A1 in spinal cord microglia is upregulated after peripheral nerve injury, however, is still unclear. Lipid rafts in glial cells play critical roles in neuroinflammatory sensitization of central pain signaling pathways by modulating the function of many ion channels and synaptic transmission.^40^, ^41^ Cholesterol efflux is inhibited to gain lipid raft support for inflammatory signaling, including in microglia.^42^, ^43^ The increased cholesterol overload in glial cells might result in increased expression of CYP7A1, which plays a key role in regulating cholesterol homeostasis and decreasing proinflammatory cytokine production.^44^

### 4.2 Mechanisms by which bile acid receptor agonists inhibit neuropathic pain

Treatments that are aimed at reducing neuroinflammation and glial cell activity, such as the use of the MAP kinase inhibitor U0126, astrocyte inhibitor fluorocitrate, and microglia inhibitor minocycline, are effective in attenuating neuropathic pain.^6^, ^45^, ^46^ Bile acids are signaling molecules that coordinately regulate metabolism and inflammation via FXR and TGR5.^9^ Consistently, our findings showed that either INT‐777 or INT‐747 inhibited the activation of glial cells in the spinal dorsal horn, and the activation of ERK, a marker of neuronal activation. Thus, it is highly possible that the activation of bile acid receptors attenuates neuropathic pain by inhibiting neuroinflammation.

Studies have shown the presence of functionally active GABA_A_ receptors on the glial cell membrane, which are extrasynaptic (δ‐containing) GABA_A_ receptors.^47^, ^48^ Neuronal excitability and synaptic transmission at the spinal dorsal horn are modulated by tonic inhibitory currents via extrasynaptic GABA_A_ receptors. We show herein that the GABA_A_ receptor δ subunit was downregulated on day 7 after SNI, suggesting that GABA_A_ receptors‐mediated tonic inhibition in the spinal dorsal horn was reduced. It has been shown that inflammation changes the GABAergic neurotransmitter system, and vice versa, GABAergic signaling can curb the inflammatory response.^49^ It is very likely that there is a positive loop between the neuroinflammatory response and reduced GABA_A_ receptors inhibition after peripheral nerve injury. Therefore, the suppression of GABA synaptic transmission after peripheral nerve injury might further potentiate the neuroinflammatory response and aggravate pain perception (Figure 11). Our present study further showed that GABA_A_ receptors antagonist bicuculline blocked the inhibitory effects of TGR5 or FXR agonists on neuropathic pain behaviors and on the activation of glial cells, which has been reported to be critical to the development and maintenance of neuropathic pain by initiating the neuroinflammatory response after peripheral nerve injury. These results suggest that bile acids exert their neuroprotective effect by modulating the function of glial cells by strengthening the inhibitory effects of extrasynaptic GABA_A_ receptors.^37^ In general, neurosteroids are potent modulators of the GABA_A_ receptors potentiating GABA‐mediated responses at low concentrations and thereby enhancing increased inhibitory tone.^50^, ^51^ TGR5, currently known as a bile acid receptor, may also act as a neurosteroid receptor in response to allopregnanolone and other neurosteroids,^22^ whose synthesis was increased in both neuronal and glial cells. Therefore, activation of bile acid receptors might ameliorate the neuroinflammatory response by potentiating GABAergic‐mediated extrasynaptic inhibition. The crosstalk of neurosteroids and bile acid receptors under the state of neuropathic pain should be examined in detail in the future.

In conclusion, the data presented herein show that activation of bile acid receptors counteracts neuropathic pain by inhibiting the activation of glial cells and neuronal sensitization, possibly by modulating GABA_A_ receptors' function.

## AUTHOR CONTRIBUTIONS

Y.W. performed the western blotting and immunohistochemistry experiments and analyzed the data. Y. Q. performed the bile acid detection experiments and the behavioral tests and analyzed the data; M.S. performed part of the behavioral tests, and critically revised the figures and the manuscript. L.X. undertook part of the behavioral tests. X. W. designed and wrote the manuscript and Q.G. revised the manuscript and helped to analyze the data. All authors reviewed the manuscript.

## FUNDING INFORMATION

This work was supported by grants from the National Natural Science Foundation (Beijing, People's Republic of China. Nos. 81870969 to X. W; Nos. 82205273 to M.S.); by the Natural Science Foundation of Guangdong Province of China (Guangzhou, People's Republic of China, Nos 2019A1515011855, 2022A1515011989 to X.W.; 2021A1515010354 to M.S.); and the Science & Technology Planning Project of Guangzhou (NO. 202102010051 to Q.G.).

## CONFLICT OF INTEREST STATEMENT

The authors declare no competing financial interests.

## Supporting information


Figure S1

## Data Availability

All data supporting the findings of this study are provided within the study and its supplementary information. Additional information will be provided upon reasonable request.
