# Supplementary material for: Activation of the bile acid receptors TGR5 and FXR in the spinal dorsal horn alleviates neuropathic pain
Source: CNS Neurosci Ther. 2023 Mar 7;29(7):1981–98. doi: 10.1111/cns.14154 (PMC10324360; doi:10.1111/cns.14154)
Supplement: Supplementary file 1 — Figure S1 [file CNS-29-1981-s001.docx]

**
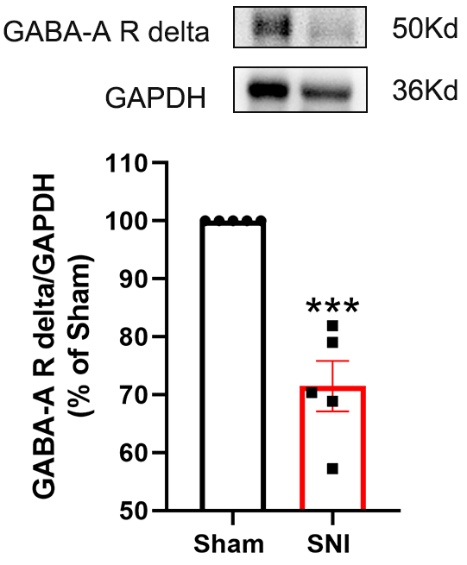
**

**Supplementary fig.1 The protein expression of GABA-A receptor delta subunit was decreased after SNI, compared to Sham group.** Protein expression levels were normalized with GAPDH and expressed as a percentage of sham levels (n=5). ***p<0.001 compared with Sham group.
